# Age-dependent development and microarchitecture of the osteochondral unit of the humeral head in harbour porpoises (*Phocoena phocoena*)

**DOI:** 10.1038/s41598-026-39726-7

**Published:** 2026-02-12

**Authors:** Marlena Maria Księżarczyk, Lonneke L. IJsseldijk, P. René van Weeren, Riccardo Levato, Jos Malda

**Affiliations:** 1 Regenerative Medicine Utrecht, Utrecht University, Utrecht, The Netherlands; 2 Department of Orthopedics, University Medical Centre Utrecht, Utrecht, The Netherlands; 3 Division of Pathology, Department of Biomolecular Health Sciences, Faculty of Veterinary Medicine, Utrecht University, Utrecht, The Netherlands; 4 Department of Clinical Sciences, Faculty of Veterinary Medicine, Utrecht University, Utrecht, The Netherlands

**Keywords:** Osteochondral unit, Aquatic mammals, Epiphysis, Humeral head, Calcified cartilage, Collagen architecture, Anatomy, Developmental biology, Evolution, Zoology

## Abstract

The body morphology of harbour porpoises *(Phocoena phocoena)*, like that of other fully aquatic mammals, differs greatly from terrestrial species due to adaptations to aquatic life. Thus, the developmental processes of the osteochondral unit (OCU) and its postnatal adaptation in harbour porpoises remain poorly understood. Here, we compared the humeral head microarchitecture in neonate, juvenile and adult harbour porpoises to better understand the relationship between environmental loading and the organisation of the OCU within diarthrodial joints. In neonates, the superficial layer of hyaline cartilage showed parallel collagen fibres, while the remaining cartilage exhibited an oblique orientation. Neither a calcified cartilage layer (CCL) nor a subchondral bone plate (SBP) was present. In juveniles the early formation of distinct zones in the articular cartilage, initial cartilage calcification and SBP formation were observed. Adults exhibited a layered hyaline cartilage structure within an arcade-like collagen arrangement, similar to terrestrial mammals. The formation of the CCL and SBP was age-dependent, as in terrestrial species, but appeared to occur later in the maturation process towards adults. These findings provide new insight into how aquatic environments shape age-related remodeling and structural adaptation of the OCU in cetacean diarthrodial joints.

## Introduction

Whales, dolphins and porpoises are fully aquatic mammals, belonging to the order of Cetacea, which includes about 90 recognised species^1^. The order is divided into Mysticeti (baleen whales) and Odontoceti (toothed whales) suborders^2^. These mammals evolved from land to water over millions of years^2^. During this transition, their body shape and organs adapted to the aquatic environment^2,3^. Evolutionary changes have intensively affected the skeletal system, especially the limbs, resulting in the loss of hind limbs and rarely leaving a residual of the pelvic bone^4^. The forelimbs evolved into pectoral fins as the bones became shorter and broader, and some of them fused, forming a largely rigid forelimb enabling swimming^2,3^. Some diarthrodial joints, like the humeral joints, retained their mobility, while others became partially or fully immobile due to cartilaginous connections between the bones’ epiphyses, which can become ossified with age^3,5^.

The formation and function of the limbs in mammals primarily takes place in the embryonic stage and is steered by prenatal gene expression^6,7^. In aquatic mammals, early embryonic cells form front and hind limb buds, but specific gene suppression limits development to the formation of only the forelimbs^2,7^. In terrestrial mammals, the final shape of the limb is refined during postnatal development, when environmental factors further influence limb morphology^8^. The postnatal development of the entire articular-epiphyseal cartilage complex has been studied extensively in terrestrial mammals, such as horses^9–11^, rabbits^12,13^, sheep^14^, and pigs^15^. The articular-epiphyseal complex is composed of the osteochondral unit (OCU) together with the epiphyseal trabecular bone in mature mammals. The OCU is formed by the articular cartilage and the underlying subchondral bone; in adult mammals there is a layer of calcified cartilage between the hyaline articular cartilage and subchondral bone in which the collagen network that forms the structural part of the hyaline cartilage is anchored^16,17^. During postnatal development, the articular cartilage transitions from an immature isotropic to a mature anisotropic organisation, materializing as the formation of an arcade-like structure, composed of collagen fibres. The development of this architecture of the collagen network is also linked to the growth of the bone epiphysis and modulated by joint loading^13^. Mechanical cues influence cartilage development and, in turn, are intimately connected to the functions of the OCU; namely shock absorption, transfer of load from cartilage to bone, and biomechanical integration^18^. However, joint loading between land and water environments differs greatly. Water is denser than air, creating more resistance when moving. On the other hand, pressure increases rapidly with depth in water, while buoyancy strongly counteracts gravitational forces in water^3,19^. Considering these significant environmental differences, it is plausible that they lead to differences in the postnatal development of the OCU. However, unlike the extensive research on bone morphology in aquatic mammals^2,20,21^, the developmental dynamics and structural characteristics of their articular cartilage have hardly been researched and therefore remain poorly understood.

A previous study already noted substantial morphological differences between the OCUs of terrestrial and aquatic mammals^22^. This interspecies comparison suggested that different loading conditions have influence on the morphological features of the OCU and highlighted that certain features of the cartilage were conserved between aquatic and terrestrial mammals where others appeared to be lost. Especially pronounced differences were observed at the cartilage-bone interface^22^. However, sample availability was limited to few individuals of different aquatic species. Species influences or the effect of ageing within a given species could therefore not be assessed^22^. Hence, it remains uncertain whether some distinct features of the terrestrial mammalian OCU are absent in aquatic mammals or if they develop later during ontogeny. To address this, we here investigate the age-dependent development of the microarchitecture of the OCU in the harbour porpoise *(Phocoena phocoena)*, a species of toothed whales. The harbour porpoise is widely distributed in the North Sea^23,24^, and in the Netherlands, a long-term post-mortem surveillance program^24^ provides access to a relatively large number of specimens from this species, across all age groups. This study specifically aims to characterize the age-related morphological differences in the humeral head of harbour porpoises, with an emphasis on the osteochondral junction (OCJ) and more specifically the orientation of the fibres of the collagen network in the extracellular matrix of the articular cartilage.

## Results

### Anatomical measurements of the pectoral fins in harbour porpoises

**Fig. 1:**
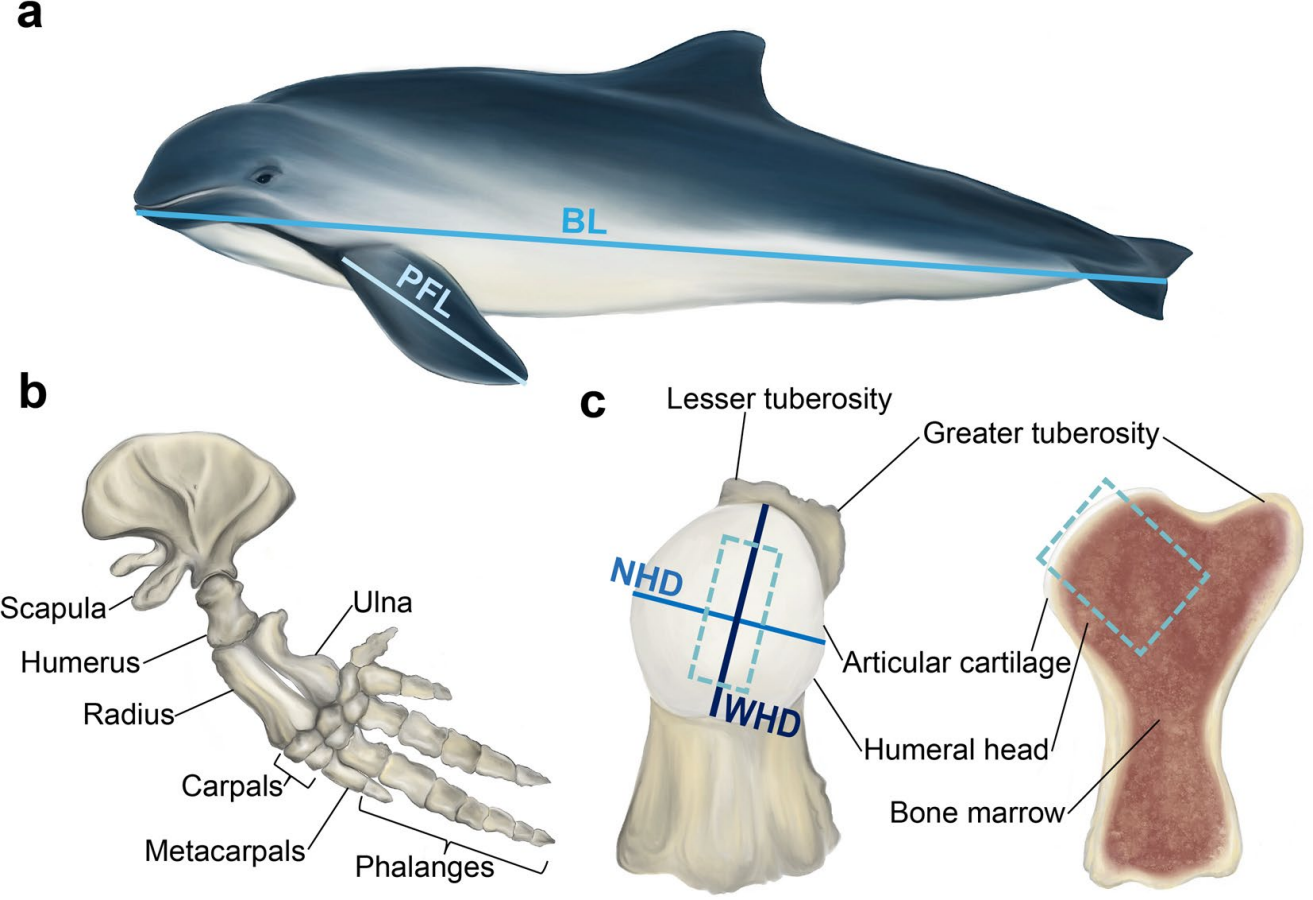
Illustration of the anatomy of the pectoral fin and humeral head depicting the measurements conducted in this study. **(a)** The entire harbour porpoise body (BL - body length; PFL - pectoral fin length). **(b)** The skeleton of the pectoral fin in adult harbour porpoises. **(c)** Anterior view of the humerus with cross-section between lesser and greater tuberosity (NHD - narrow humeral head diameter; WHD - wider humeral head diameter; WHD was measured along the medial-lateral axis). The dashed line indicates the material collected for histological analysis.

**Fig. 2:**
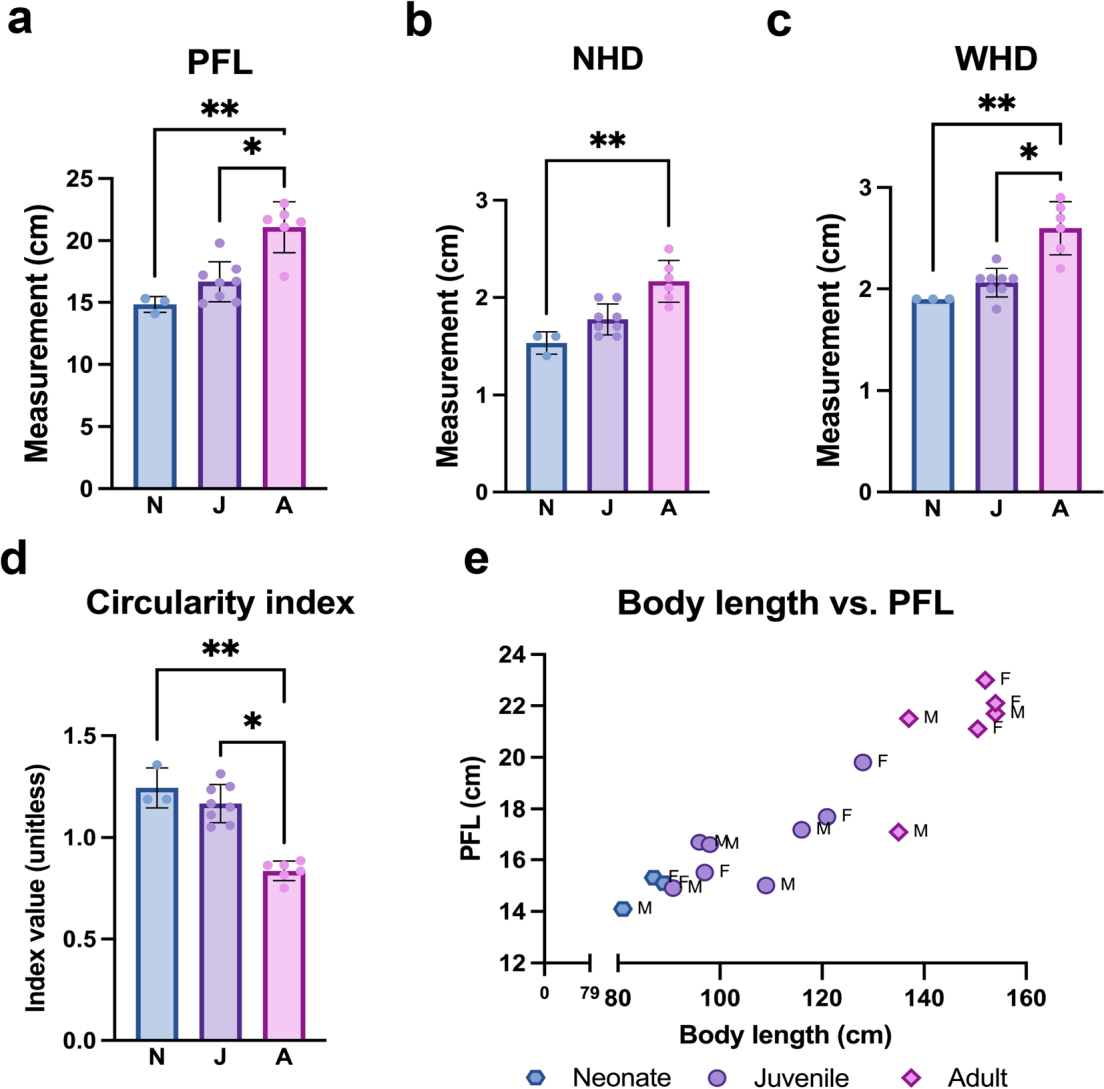
Anatomical measurements in neonate (N), juvenile (J) and adult (A) harbour porpoises. **(a)** Pectoral fin length (PFL). **(b)** Narrow humeral head diameter (NHD). **(c)** Wider humeral head diameter (WHD). **(d)** Humeral head circularity index (WHD/NHD ratio). Significance is indicated as *p < 0.05; **p < 0.01. **(e)** Relationship between body length and pectoral fin length. Each point represents an individual animal. F=female, M=male.

The pectoral fin (Fig. 1a) in all examined harbour porpoises consists of the scapula, humerus, radius, ulna, carpals, metacarpals and phalanges, which are illustrated in Fig .1b. The proximal epiphysis of the humerus of the examined harbour porpoises features a well-defined humeral head with a greater and lesser tuberosity (Fig. 1c). The pectoral fin length (PFL), narrow humeral head diameter (NHD), and wider humeral head diameter (WHD) size varied and increased with age within the examined groups, as reflected in Fig. 2a-c. The humeral head has an oval shape and becomes progressively less circular as indicated by the decreasing circularity index value (Fig. 2d), reflecting age-related shape remodeling. Body length and pectoral fin length across individuals increased with age as presented in Fig. 2e.

### Osteochondral unit development

**Fig. 3:**
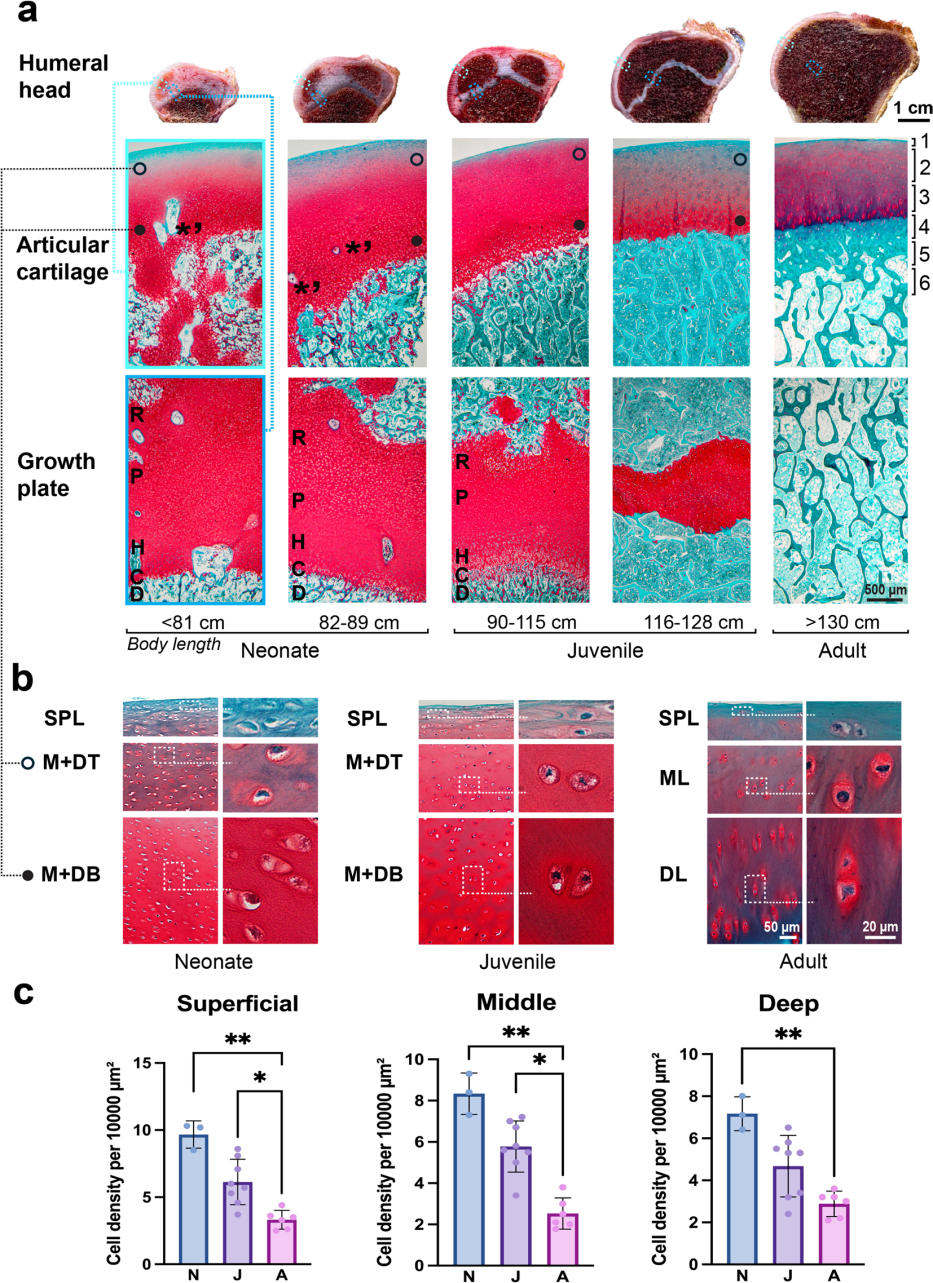
**(a)** Age-related development of the OCU in the humeral head of the harbour porpoise. Top row: macroscopic images of a cross-section of the humeral head in the three age groups: neonates, juveniles, and adults; articular cartilage is marked with a light blue dashed rectangle and the growth plate with a dark blue dashed rectangle. Middle and lower row: histological sections stained with Safranin-O (red) and Fast Green (green). In the middle row, asterisks with apostrophes indicate cartilage canals. In neonates and juvenile porpoises, in the middle row the open-black circle indicates the top of the ‘middle + deep’ regions (M + DT) of articular cartilage, and the filled black circle indicates the bottom (M + DB). Numbers in middle row indicate the different layers of the OCU in adults: 1 - superficial layer, 2 -middle layer, 3 - deep layer, 4 - calcified layer, 5 - subchondral bone plate, and 6 - subchondral trabecular bone. In the lower row, letters indicate the different layers of the growth plate: R - zone of resting chondrocytes, P - zone of cell proliferation, H - zone of cell hypertrophy, C - zone of calcification, and D - zone of bone deposition. **(b)** Morphology of the lacunae of the cartilage and chondrocytes (right panels), selected from the areas marked by a dashed rectangular outline on the left panels; in neonate and juvenile harbour porpoises images were captured from the superficial layer (SPL) and the M + DT and M + DB, and in adults from the SPL, middle layer (ML), and deep layer (DL). **(c)** Average cell density per 10,000 µm^2^. In neonate and juvenile porpoises, the results counted from the M + DT are presented in the ‘Middle’ chart, and the results of M + DB are presented in the ‘Deep’ chart. In adult harbour porpoises, results are counted in the three examined layers; (*p < 0.05; **p < 0.01).

Large morphological differences were observed in the articular-epiphyseal cartilage-bone complex of the humeral head between the different age groups (Fig. 3a). The average thickness of the entire layer of articular cartilage was greatest in neonates (1.49 ± 0.21 mm), was less in juveniles (1.39 ± 0.19 mm) and further decreased in adults (1.26 ± 0.14 mm). This gradual numerical decline did, however, not result in statistically significant differences between the age classes, most likely because of group size. The growth plate was clearly present in neonates (2.29 ± 0.12 mm), partially resorbed in juveniles (1.34 ± 0.53 mm) and fully closed and replaced by trabecular bone in adults. This developmental process led to significant differences between groups neonate vs. adult and juvenile vs. adult (*p* < 0.001). In neonate and juvenile porpoises with an open or partially resorbed growth plate, chondrocytes were arranged in a well-defined columnar arrangement. In contrast, in the end stage of closing the growth plate, this columnar arrangement was no longer observed.

In neonates, a zonal organisation of the articular cartilage was not yet distinguishable. The superficial cartilage layer was in an early stage of formation but with less defined layer boundaries than in juvenile or adult porpoises, whereas the remaining cartilage appeared relatively homogeneous. Additionally, cartilage canals containing the vascular structures of the epiphyseal cartilage associated with the early stage of the process of endochondral ossification, were present throughout the entire thickness of the articular cartilage (Fig. 3a).

In juvenile porpoises, the articular cartilage exhibits a clearly defined superficial layer and shows early development of the zonal organisation of the middle and deep layers. Individuals in this age group range from 90 cm to 128 cm in body length. Upon closer examination of the OCJ, morphological differences were observed between sub-groups of shorter and longer animals. In juveniles measuring 116–128 cm, the OCJ with subchondral bone was clearly identifiable, whereas in the smaller specimens (90–115 cm), the junction displayed irregular boundaries. In all examined juveniles, a distinct subchondral bone plate (SBP) was absent (Fig. 3a).

In adults, the zonal organization of the cartilage could be divided into four distinct layers: superficial, middle, deep, and calcified (Fig. 3a). Moreover, this was the only group in which a SBP was present, with a mean thickness of 0.50 ± 0.22 mm. Cartilage remnants were abundant in the trabecular bone of neonatal epiphyses, less frequent in juveniles, and absent in adults.

In the three examined age groups, the chondrocytes in the superficial layer exhibited a flattened morphology. In neonate and juvenile porpoises, the cartilage beneath the superficial layers contained predominantly rounded chondrocytes, whereas in adults, such round cells were confined to the middle layer. In the deep layer of the adults, the chondrocytes were vertically aligned relative to the cartilage-bone interface and displayed an elongated oval shape (Fig. 3b). The average cell density was the highest in neonates in the three selected areas, and in juveniles average cell density was decreased, and the lowest number of cells was counted in adults (Fig. 3c). Significant differences in cell density were detected among the examined groups (Fig. 3c).

### Formation of the calcified cartilage layer (CCL)

**Fig. 4:**
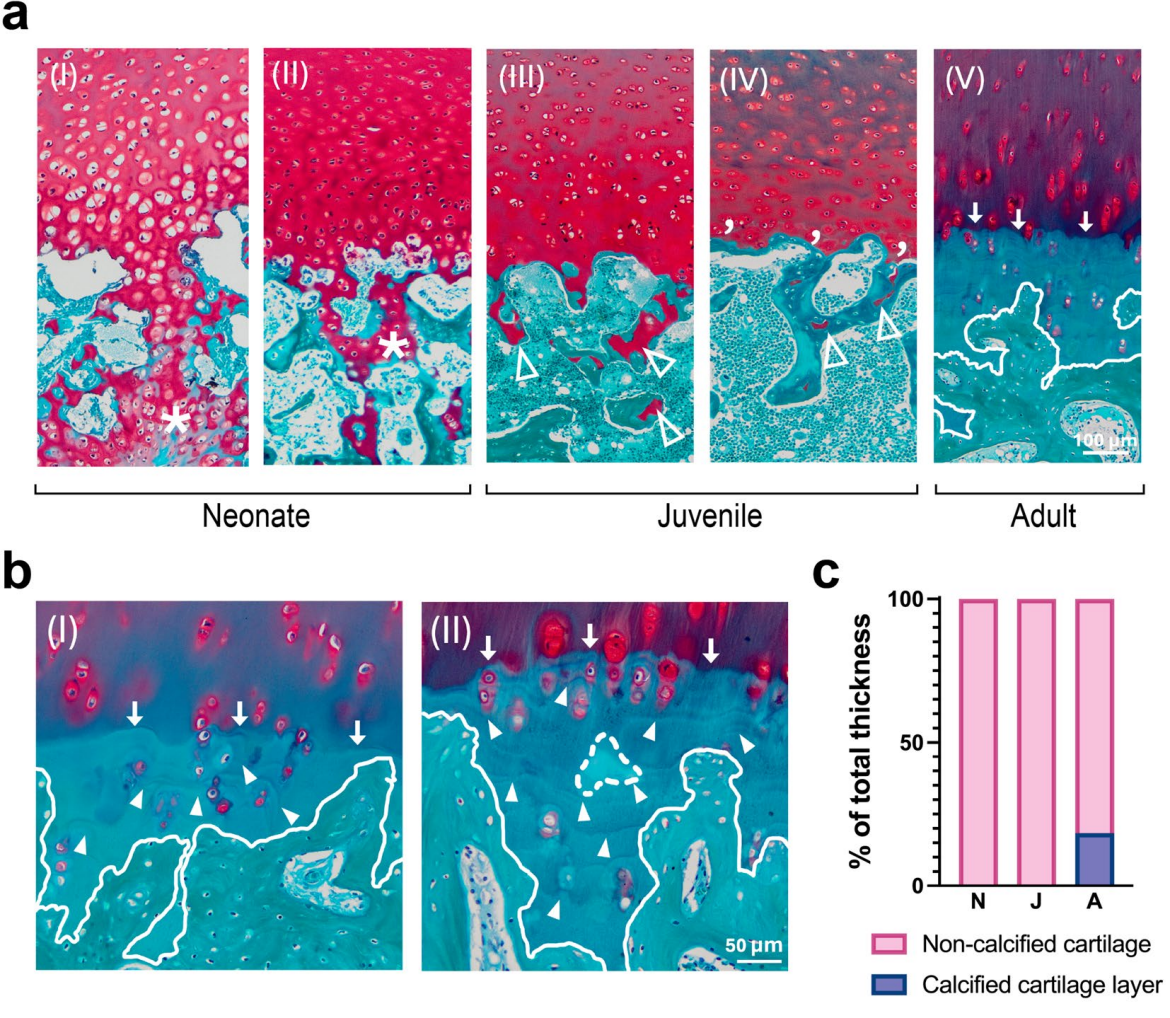
**(a)** Histological images of the cartilage–bone junction in the three age groups. In neonates, the secondary ossification center is marked with an asterisk (a.I and a.II). In juveniles, the open arrowheads indicate cartilage patches between ossified areas (a.III and a.IV). Moreover, visible mineralized deposits surround the chondrocytes located above the cartilage-bone junction (apostrophe). The white line in Fig. 4a.V indicates the borders of the formed calcified cartilage layer with the subchondral bone and arrows mark the tidemark. **(b)** Arrowheads represent the calcification line in the calcified cartilage layer; arrows mark the tidemark, and dashed lines indicate subchondral bone fragments within the calcified matrix. **(c)** The percentage of the total average thickness of non-calcified and calcified cartilage layers in neonate, juvenile, and adult porpoises was measured.

It was also observed that the formation of the CCL in harbour porpoises is an age-dependent process (Fig. 4). In neonates the CCL was completely absent, reflecting skeletal immaturity. A fully developed CCL was also absent in juveniles, although minimal deposition of calcified material was observed around the chondrocytes near the OCJ in some larger juveniles. In adults, the CCL was present, but irregularly structured with non-uniform morphology, often containing subchondral bone fragments embedded with the calcified matrix. The average thickness of the CCL compared to non-calcified cartilage of all examined age groups was expressed as percentage of the total thickness and presented in Fig. 4c. The subchondral bone boundary was undulating, and the CCL closely conforms to its contours. In adults, hypertrophic chondrocytes were sparsely distributed within the CCL matrix. All adult individuals exhibited a well-defined tidemark at the interface with the non-calcified cartilage. The tidemark was less undulating compared to the boundary of the CCL with the subchondral bone.

### Collagen fibre orientation of the articular cartilage

**Fig. 5:**
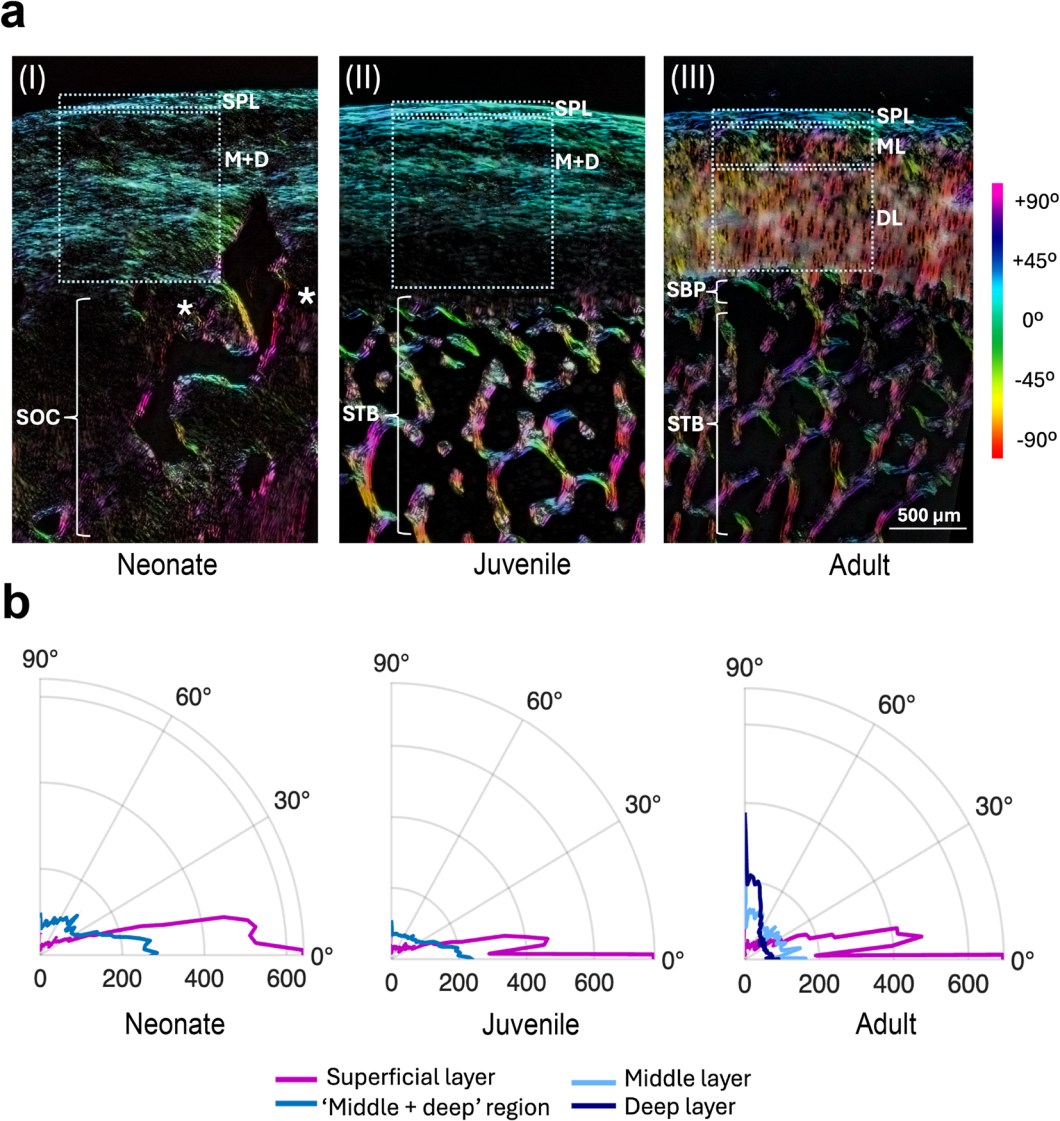
**(a)** Orientation colour map merged from three PLM images taken at 0°, 45°, and 90° angles on a rotated polarizer, demonstrating collagen fibres orientation in the three examined groups. In a.I and a.II rectangles show the selected ares where the measurements of the collagen fibre distribution were taken in the superficial layer (SPL) and ‘middle + deep’(M + D) region and in a.III in the SPL, middle layer (ML) and deep layer (DL). Asterisks in (a.I) indicate collagen fibres located between the secondary centers of ossification (SOC) predominantly arranged perpendicular to the surface. In a.III SBP means subchondral bone plate and STB subchondral trabecular bone. **(b)** Polar plots of collagen fibre distribution in the superficial and M + D regions in neonates and juveniles, and the superficial, middle, and deep layers in adults. 0° on the x-axis represents fibres parallel to the surface, while 90° on the y-axis indicates fibres perpendicular to the surface. The values on the x-axis indicate how strongly fibres are aligned as orientation intensity.

Polarized Light Microscopy (PLM) analysis showed that collagen fiber orientation differs across the examined age groups (Fig. 5). In neonate, juvenile, and adult harbour porpoises, the collagen fibres in the superficial layer were oriented parallel to the articular surface. In neonate and juvenile porpoises, the collagen fibres of the remaining articular cartilage, shown as ‘middle + deep’ (Fig. 5), were organised with a predominance of fibres oriented more oblique (between 0° and 45°) relative to the surface (Fig. 5a and b). Notably, in neonates, collagen fibres located between the secondary centres of ossification are predominantly arranged perpendicular to the surface. In adults, cartilage exhibits a fully developed four-layered structure as in terrestrial mammals with a clearly visible arcade-like collagen structure. Within the middle layer, collagen fibres remain randomly organised, whereas in the deep layer, the fibres are oriented perpendicular, creating a palisade-like appearance.

### Biochemical analysis of DNA, glycosaminoglycan, and collagen content

**Fig. 6:**
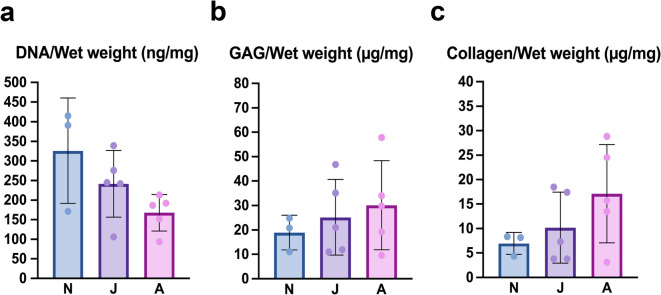
Average **(a)** DNA, **(b)** glycosaminoglycan (GAG), and **(c)** collagen content of the hyaline cartilage collected from neonate (N), juvenile (J), and adult (A) harbour porpoises.

During postnatal development in all examined groups, a decrease in DNA content was observed (Fig. 6a), which is in line with the average cell density presented in Fig. 3c. The glycosaminoglycan (GAG)(Fig. 6b) and collagen (Fig. 6c) content increased across the three examined age groups. For DNA, GAG, collagen, changes followed a similar developmental trajectory but were not statistically significant (Fig. 6).

## Discussion

Here, we describe age-group-related postnatal developmental changes of the humeral head in harbour porpoises. Despite numerous studies on the postnatal development of the epiphysis of long bones in terrestrial species^12,15,25^, this is the first study on the postnatal development of the epiphysis in aquatic species, with particular interest in the OCU, using macroscopic assessment and histological and biochemical analyses.

Neonate porpoises exhibit morphological similarities with terrestrial species in the developing epiphysis of the bone, with a well-defined secondary centre of ossification^10,26^. In the epiphysis of neonates from terrestrial species and harbour porpoises, an open growth plate indicates skeletal immaturity, and the columnar arrangement of chondrocytes in the growth plate indicates longitudinal growth^27,28^. During the maturation of the porpoise’s humeral epiphysis, we can observe a gradual endochondral ossification of the growth plate, similar to that in terrestrial animals^13,27^. In terrestrial species, endochondral ossification is driven by hormonal and molecular regulations, phylogenetic control, and mechanical loading. The growth plate closure patterns in terrestrial animals follow common trends across different mammalian groups, the timing of this closure pattern varies among species and is related to phylogenetic variation^29^. In this study, we focus on the histological features of the growth plate closure across three age groups. The phylogenetic, hormonal, and molecular aspects in aquatic species remain unclear, and a more detailed study could provide further insight into this process.

In mature porpoises, the OCJ exhibited a well-defined SBP and a CCL, which has not been described in previous studies of this species^22^. This discrepancy may be related to small numbers of individuals in previous studies and the lack of detailed age group classifications between late-stage juveniles and adult harbour porpoises. In adults, the CCL appeared irregular in histology sections, often containing subchondral bone fragments within the calcified cartilage matrix. This is most likely due to the highly irregular three-dimensional configuration of the CCL-subchondral bone interface. The morphology of the adult harbour porpoises’ OCJ differs from that of terrestrial species described in earlier reports, where this interface has a more regular, straight configuration^22,30^. The regular formation of the CCL in terrestrial animals results from compressive loads on the joint during movement^31^. This shape helps to facilitate the transition between hyaline cartilage and a rigid subchondral bone by distributing stress relatively evenly across the joint surface to prevent cartilage detachment^31^. However, loading conditions differ greatly between aquatic and terrestrial mammals. Unlike in the terrestrial environment, the marine environment reduces the forces acting on the joint^3,32^. Therefore, the irregular shape of the CCL in harbour porpoises likely results from differences in loading caused by very differing environmental conditions.

Notably, significant morphological differences exist in the zonal organisation of the articular cartilage between terrestrial animals and harbour porpoises during the juvenile stage^26,33^. For example, in rabbits, the pattern of collagen arcades is established much earlier, within about 6 weeks^12,33^ and the fully developed zonal organisation of articular cartilage is completely established by 12–14 weeks of age^33^. In sheep, ossification of OCJ and the cartilage zonation becomes detectable at 8 weeks, reaching complete division by approximately 40 weeks^34^. In horses, the zonal organisation of the articular cartilage begins around 4 months of age^35^; by 4.5 months, a parallel collagen arrangement is evident, which gradually develops into a fully Benninghoff-like arcade structure over the next 10 months^11^. In our study, animals were classified as neonates, juveniles, and adults based on body length, following the standard method for age group determination in this species^24^. Consequently, it was not possible to assign the exact age in years corresponding to the time when the zonal organisation with arcade-like collagen structures is fully developed. However, in harbour porpoises, the CCL with fully zonally organised cartilage and characteristic collagen arcade structures does not appear until adulthood, indicating that cartilage maturation occurs considerably slower than in terrestrial species, where such organisation occurs relatively early in life^36^. Developmental changes in the articular cartilage involve not only the zonal organisation and orientation of collagen fibres, but also the biomechanical and biochemical properties^14^. In harbour porpoises, the developmental trajectories of collagen, GAG, and DNA compositions closely mirror the biochemical changes reported in terrestrial species^37^.

A complex interplay of many factors regulates joint formation during postnatal development^6,8^. An important factor responsible for shaping the final appearance of adult articular cartilage during postnatal development is loading^18,38^. Loading is also closely connected to maintenance of homeostasis within the joint, balancing extracellular matrix (ECM) integrity and the mechanotransduction capacity of the developing tissue^39,40^. It has, for example, been shown that cyclic compression caused by loading and joint movement enhances the interstitial fluid flow and hydrostatic pressure gradients inside the cartilage matrix, which promotes better nutrition of the entire cartilage^41,42^. Proper diffusion of nutritional components is essential to synthesize the ECM components in growing cartilage^43^.

Functional adaptation of the joint to weight-bearing occurs early in life. For example, in horses, regular early-life training can enhance articular tissue quality over time and support long-term joint health^9,25^. In a later study, it was reported that Benninghoff’s classic perpendicular collagen arcades in horses do not consistently appear as an architectural feature, but instead adapt dynamically to local mechanical forces^36^. Furthermore, exercise promotes greater collagen parallelism and modifies fibre orientation^36^. In foals deprived of early-life exercise, ECM development was markedly delayed, unlike in the exercised group^44^. Moreover, joint immobilization can further disrupt or delay the normal process of formation of the articular ECM^45^. Improper cartilage maturation during joint morphogenesis can result in dysplasia or early degeneration, as is well documented for horses^10,46^. Although the relationship between postnatal development and the onset of osteochondral diseases has not yet been examined in aquatic species, there is anecdotal evidence of only a few cases of joint dysplasia and osteoarthritis in these species^47^. Furthermore, it remains unclear whether the morphological differences observed during epiphyseal formation in aquatic mammals are unique to the harbour porpoise or if they represent a broader pattern across marine mammals, potentially involving species-specific variation in cartilage zonal organisation. While our study provides the first description of the postnatal epiphyseal development in harbour porpoises, it was conducted post-mortem, which limited our ability to assess functional adaptation and long-term osteochondral maturation in joints of healthy aquatic species. Further studies are necessary to understand how mechanical loading and aquatic locomotion affect joint development in aquatic animals.

Considering the morphological characteristics observed during postnatal development of the OCU in harbour porpoises, our findings contribute to a more comprehensive understanding of load-induced joint formation. This knowledge enhances our understanding of the physiological processes underlying the growth of healthy joints in aquatic mammals exposed to mechanical stimulation and informs the evaluation of regenerative medicine approaches aimed at osteochondral repair. A more precise understanding of osteochondral microarchitecture formation will support the development of improved strategies from ECM restoration, which in turn could enhance biofabricated osteochondral constructs designed to withstand diverse loading conditions and facilitate efficient joint movement.

## Conclusion

This is the first report describing the postnatal age-related development of the OCU in aquatic mammals. While the process shows strong similarities with that observed in terrestrial mammals, the typical Benninghoff collagen arcade structures do not form until adulthood, in contrast to terrestrial species. This indicates that due to the distinct mechanical loading environments, the development of the articular cartilage tissue in aquatic species appears to progress more slowly compared to that in terrestrial species. These findings are essential for understanding age-dependent remodeling and mechanical adaptation of the OCU in aquatic species. They also provide a foundation for applying this knowledge to regenerative medicine by elucidating how mechanical loading contributes to articular cartilage biology, tissue maturation, and evolutionary morphological changes.

## Materials and methods

### Sample collection

Samples were harvested post-mortem from the left humeral head during necropsies, which are routinely conducted as part of a long-term monitoring program at the Division of Pathology, Faculty of Veterinary Medicine, Utrecht University in the Netherlands^24^. During necropsies, the state of decompositions was judged, and animals with signs of decomposition (like sloughing of the skin and of the entire joint capsule with shrinking of the surrounding tissue) were excluded, as were animals demonstrating signs of cartilage degeneration. Ultimately, seventeen harbour porpoises without signs of degeneration or decomposition were included in the study. Of these, sixteen harbour porpoises were stranded and found dead or died shortly after stranding on the Dutch North Sea coast, and one was born and has lived under human care. As the death of these animals was not related to animal experimentation, further ethical approval was not required. The performed measurements during the post-mortem examination are shown in Fig. 1; Table 1. The shoulder joint was selected for the study, as this joint has retained mobility during the evolutionary changes in aquatic mammals after they returned to the sea while the remaining joints distal to the humeral bone became less mobile and fused (Fig. 1b). Upon collection, the whole humeral bone and its cross-section were macroscopically photographed. Samples of the OCU, approximately 1.5 cm in width and 3.5 cm in thickness, were dissected from the central loaded area of the joint surface (Fig. 1c). Samples from all harbour porpoises were fixed in 4% formalin for further histology analysis. Non-fixed samples from 13 harbour porpoises were stored in PBS (-20 °C) for biochemical assays until further analysis was performed.

**Table 1:** Body measurements of 17 harbour porpoises in different age groups. F=female; M=male; N=neonate; J=Juvenile; A=Adult.

Harbour porpoise No.	Sex	Age group	Weight (kg)	Body length (cm)	Pectoral fin length (cm)
1.	M	N	7.5	81.0	14.1
2.	F	N	12.7	87.0	15.3
3.	F	N	15.0	89.0	15.1
4.	M	J	9.3	90.8	14.9
5.	M	J	18.3	96.0	16.7
6.	F	J	14.3	97.0	15.5
7.	M	J	13.4	98.0	16.6
8.	M	J	17.9	109.0	15.0
9.	M	J	21.2	116.0	17.2
10.	F	J	32.0	121.0	17.7
11.	F	J	28.3	128.0	19.8
12.	M	A	36.7	135.0	17.1
13.	M	A	29.0	137.0	21.5
14.	F	A	48.0	150.5	21.1
15.	F	A	39.6	152.0	23.0
16.	M	A	48.5	154.0	21.7
17.	F	A	60.8	154.0	22.1

### Age group division

The age group division of the porpoises was carried out based on body length, with the final differentiation between juveniles and adults made based on gonadal appearance assessed during necropsies^24,48^. The examined individuals ranged in length from 81 cm to 154 cm and were divided into three groups: neonate (< 90 cm, *n* = 3), juvenile (90 –128 cm, *n* = 8) with sub-groups division due to morphological differences of the OCJ as smaller juveniles (90–115 cm, *n* = 5) and larger juveniles (116–128 cm, *n* = 3), and adult (> 130 cm, *n* = 6). Quantitative analyses were performed only on the three-group division as neonate, juvenile and adults, whereas the subgroups of the juvenile porpoises were used for qualitative morphological observation only. Detailed information of the length of animals is presented in Table 1. Sex was not considered as a grouping factor in this study.

### Histology

After 7 days of fixation, samples from 17 harbour porpoises were transferred to a decalcification solution (Luthra solution; 3.2% 11 M HCl with 10% formic acid in distilled water) and processed as previously described by Mancini et al.^22^. Embedded in paraffin samples were cut into 5 μm histological sections and stained with Hematoxylin & Eosin and Safranin-O & Fast Green staining to visualize cartilage and growth plate microarchitecture. For the collagen fibre orientation evaluation, sections were stained with Picrosirius Red & Weigert’s Hematoxylin.

### Polarized light microscopy

The Picrosirius red-stained histological slides were viewed under a microscope (Olympus BX43, Olympus) with a graduated rotatable analyzer (U-AN360P, code 037862, Olympus). Images were acquired (under 1.25x magnification) using a digital camera (Olympus DP73) connected with Cell^F image analysis software (Olympus, USA). Three PLM images captured under 0°, 45°, and 90° degrees from the same region were merged and analyzed using FIJI software with the OrientationJ plugin for an orientation color map. The orientation intensity values obtained from OrientationJ are dimensionless and represent a relative measure of fiber alignment derived from image-based orientation analysis. The PLM measurements were performed as previously described by Cunniffe et al.^49^. The collagen orientation regions of interest (ROI) were determined from PLM images, with additional analysis of the same picrosirius-red stained sections captured under light microscopy to assess different articular zones by chondrocyte distribution and shape across each layer. Due to the immature organization of cartilage layers in neonate and juvenile samples, only two distinguishable ROIs were identified based on visible cartilage morphological boundaries as superficial layers, and below, the ‘middle + deep’ region of interest. In adult individuals, ROI were separated into three layers: superficial, middle, and deep. Collagen fibre orientation data were processed and visualized in MATLAB (MathWorks, USA) using a polar plot to generate pole figures illustrating the angular collagen fibre distribution.

### Biochemical analysis of DNA, glycosaminoglycan, and collagen content

Cartilage samples containing the superficial, middle and deep layer only were weighed before analysis (neonate, *n* = 3; juvenile, *n* = 5; adult, *n* = 5). Then, samples were digested overnight at 60 °C in 500 µL of papain solution containing 0.01 M cysteine, 250 µg/mL papain, 0.2 M NaH_2_PO_4_, and 0.01 M EDTA.2H_2_O. Afterwards, the samples were vortexed and digested for 1 h at the same temperature. The digested samples were then divided for specific assays. The PicroGreen DNA assays using Quant-iT™ PicoGreen^®^ dsDNA (Invitrogen, P7589)^50^ were used to measure DNA content. The dimethylmethylene blue (DMMB) assay^51^ was employed to quantify glycosaminoglycan (GAG) content. Collagen content was analysed using a hydroxyproline assay because hydroxyproline is mainly found in collagen, making this assay an indicator of collagen content. Papain-digested samples were hydrolysed overnight at 108 °C in 100 µL of 0.4 M NaOH, after which 100 µL of 1.4 M citric acid was added. Then, samples were centrifuged and pipetted into standard 96-well plates, then incubated for 20 min at room temperature with chloramine-T reagent (MERCK, 2426) dissolved in stock buffer containing 0.24 M citric acid, 0.88 M CH_3_COONA.3H_2_O, and 0.85 M NaOH in RX-water, pH 6.1 (pH determined by adding Acetic acid). Then followed by Dimethylaminobenzoaldehyde reagent (MERCK, 3058) for 20 min at 60 °C. Absorbance was read at 570 nm using a microplate reader (Clariostar^®^, BMG Labtech).

### Data and statistical analysis

The histological description of the epiphysis morphology was based on the analysis of three histological sections per staining from the central loaded region of the humeral head of all examined animals. The average thickness of the OCU compartment and growth plate was determined by averaging five measurements per figure, taken from a randomly selected region of the histological sections. All thickness measurements were calculated using the ImageJ software. The thickness results were presented as the mean ± standard deviation. Statistical analyses were preformed using GraphPad Prism 10.0 (GraphPad Software, San Diego, CA, USA). The non-parametric statistical analyses used a Kruskal-Wallis test with Dunn’s post hoc analysis for all data in this study. The level of significance was set at *p* < 0.05.

### Cell density

Cell density was calculated as the number of cells per unit area (cells/µm²) and measured using *ImageJ* software with the grid plugin on histological sections stained with Safranin-O & Fast Green staining. The grid area per point was set at 10,000 μm² (each square measuring 100 × 100 μm). Cells were counted from three distinct cartilage regions in adult specimens with zonal organization: superficial, middle, and deep layers. In neonates and juveniles, cells were counted from the superficial layer and a combined ‘middle + deep’ region due to the lack of distinct zonal separation between the middle and deep cartilage layers. In the ‘middle + deep’ region of interest, cells from the top of the middle area were designated as the middle region, while those from the bottom near the OCJ were identified as the deep region. Ten squares for each cartilage layer or region were randomly selected for manual counting. The average cell count for each cartilage layer or region was presented as the mean ± standard deviation.

## Data Availability

The original contributions presented in the study are included in the article. Further inquiries can be directed to the corresponding author.
